# Environmental risk factors and changing spatial patterns of human seropositivity for *Echinococcus* spp. in Xiji County, Ningxia Hui Autonomous Region, China

**DOI:** 10.1186/s13071-018-2764-1

**Published:** 2018-03-09

**Authors:** Angela M. Cadavid Restrepo, Yu Rong Yang, Donald P. McManus, Darren J. Gray, Tamsin S. Barnes, Gail M. Williams, Ricardo J. Soares Magalhães, Archie C. A. Clements

**Affiliations:** 1 Research School of Population Health, The Australian National University, Canberra, Australian Capital Territory 0200 Australia; 2 Ningxia Medical University, 692 Shengli St, Xingqing, Yinchuan, Ningxia Hui Autonomous Region China; 3 Molecular Parasitology Laboratory, QIMR Berghofer Medical Research Institute, Brisbane, Queensland 4006 Australia; 4 The University of Queensland, School of Veterinary Science, Gatton, Queensland Australia; 5 The University of Queensland, Queensland Alliance for Agriculture and Food Innovation, Gatton, Queensland 4343 Australia; 6 The University of Queensland, School of Public Health, Brisbane, Queensland 4006 Australia; 7 Children’s Health and Environment Programme, Queensland Children’s Medical Research Institute, The University of Queensland, Brisbane, Queensland 4101 Australia

**Keywords:** Human echinococcoses, *Echinococcus granulosus*, *Echinococcus multilocularis*, Environment, Geographical information systems, Remote sensing, Xiji County, Ningxia Hui Autonomous Region

## Abstract

**Background:**

Human echinococcoses are parasitic helminth infections that constitute a serious public health concern in several regions across the world. Cystic (CE) and alveolar echinococcosis (AE) in China represent a high proportion of the total global burden of these infections. This study was conducted to predict the spatial distribution of human seropositivity for *Echinococcus* species in Xiji County, Ningxia Hui Autonomous Region (NHAR), with the aim of identifying communities where targeted prevention and control efforts are required.

**Methods:**

Bayesian geostatistical models with environmental and demographic covariates were developed to predict spatial variation in the risk of human seropositivity for *Echinococcus granulosus* (the cause of CE) and *E. multilocularis* (the cause of AE). Data were collected from three cross-sectional surveys of school children conducted in Xiji County in 2002–2003, 2006–2007 and 2012–2013. Environmental data were derived from high-resolution satellite images and meteorological data.

**Results:**

The overall seroprevalence of *E. granulosus* and *E. multilocularis* was 33.4 and 12.2%, respectively, across the three surveys. Seropositivity for *E. granulosus* was significantly associated with summer and winter precipitation, landscape fragmentation variables and the extent of areas covered by forest, shrubland, water and bareland/artificial surfaces. Seropositivity for *E. multilocularis* was significantly associated with summer and winter precipitations, landscape fragmentation variables and the extent of shrubland and water bodies. Spatial correlation occurred over greater distances for *E. granulosus* than for *E. multilocularis.* The predictive maps showed that the risk of seropositivity for *E. granulosus* expanded across Xiji during the three surveys, while the risk of seropositivity for *E. multilocularis* became more confined in communities located in the south.

**Conclusions:**

The identification of high-risk areas for seropositivity for these parasites, and a better understanding of the role of the environment in determining the transmission dynamics of *Echinococcus* spp. may help to guide and monitor improvements in human echinococcosis control strategies by allowing targeted allocation of resources.

## Background

Cystic echinococcosis (CE), caused mainly by infection with *Echinococcus granulosus*, and alveolar echinococcosis (AE), caused by infection with *E. multilocularis*, are chronic and potentially fatal diseases that have a wide geographical distribution across the world.

According to global estimates, the number of new cases of CE is 188,000 every year, which represents a human health burden of 184,000 disability adjusted life years (DALYs) [1]. There are 18,235 new AE cases annually, which result in approximately 666,433 DALYs lost [2].

China is a country affected heavily by human echinococcoses [3]. In China, the nationally estimated numbers of CE and AE cases explain 40 and 95% of the total global burden of the infections, respectively [2, 4]. The second survey of parasitic diseases conducted in China in 2001–2004 found that approximately 380,000 people were affected by these two types of echinococcoses, and 50 million were at risk of infection nationwide [5]. Prevalence of CE and AE was particularly high in seven provinces/autonomous regions located in Western China: Qinghai, Gansu, Sichuan, Xinjiang Uighur Autonomous Region (AR), Tibet AR, Ningxia Hui AR and Inner Mongolia AR [6, 7]. However, regional and local variation in echinococcosis risk is high, with the diseases being particularly prevalent among poor pastoral minority groups [2, 8, 9].

The National Control Programme to prevent and cure echinococcoses in China was developed by the National Health and Family Planning Commission (formerly the Ministry of Health) in 2005 [6]. To date, applying and sustaining the programme has proven difficult in most endemic regions due to the lack of effective surveillance data, dispersed populations and movement of people and livestock to summer pastures [10]. Screening surveys to detect early cases are primarily conducted in the most-affected regions of China [6, 11]. Therefore, the national prevalence reports may be biased [10, 11]. Because human echinococcoses are characterised by long incubation periods that precede clinical diagnoses, current epidemiological estimates may be overlooking the relative contribution of asymptomatic or undiagnosed/untreated CE and AE cases [10]. Consequently, better surveillance and response tools are required to estimate and predict the real impact of these two diseases in China, and to strengthen the implementation of prevention and control interventions in targeted high-risk areas [12].

*Echinococcus granulosus* is primarily maintained in life-cycles that involve domestic animals, while *E. multilocularis* is typically a wildlife parasite [13]. Both species are transmitted in multi-host systems that are determined by factors that govern the presence/absence and infectivity of the parasites and also the population dynamics and interactions of the hosts [13]. Thus, special emphasis is currently being placed on identifying the role of environment factors in influencing the transmission patterns of *E. granulosus* and *E. multilocularis* and explaining the apparent emergence and re-emergence of human infections in several regions of the world [14–18]. The Chinese government is implementing a series of extensive landscape regeneration projects to restore the country’s degraded ecological landscape [19, 20]. Studies conducted in various echinococcosis-endemic regions have documented that land cover transformations are related to higher population densities of key intermediate hosts for *E. multilocularis*, which has increased the risk of human AE infection [21–28]. Hence, research also needs to be conducted to better describe the ecological processes that may lead to variations in the transmission patterns of *E. granulosus* and *E. multilocularis* based on shifting environmental factors [29]. This information will be essential to monitor emergence or re-emergence of the transmission of both parasites [29].

Bayesian model-based geostatistical approaches have been increasingly used in research focused on characterising the geographical patterns of infectious diseases and quantifying their associations with potential risk factors [1, 30–33]. Model-based geostatistics incorporates a model of the spatial correlation structure of the data with the effect of covariates to predict a variable of interest (e.g. seropositivity for *Echinococcus* spp.) in unsampled locations, and to quantify the associated uncertainty in the estimated parameter values [34]. These methods provide a valuable and flexible framework that can be used to support the process of decision-making during the implementation of a control programme [34].

Using Bayesian model-based geostatistics, we aimed to explain the spatiotemporal distribution of human seropositivity for *E. granulosus* and *E. multilocularis* in Xiji County, Ningxia Hui Autonomous Region (NHAR), China, based on selected environmental factors. In the study, the term human seropositivity was meant to signify that children harboured possibly the metacestode stage of *E. granulosus* and/or *E. multilocularis*, whether or not they had evidence of active cyst(s) in the abdominal ultrasound or any manifestation of disease (following the description of a possible echinococcosis case suggested elsewhere [35]. Also, we aimed to produce spatial prediction maps to show the evolving geographical distribution of seropositivity for these parasites species at three different time points during the last decade. These maps will be useful to inform decisions on where communities at high risk of echinococcoses are located in China, and to help prioritise and target resources for prevention and control.

## Methods

### Study area

**Fig. 1:**
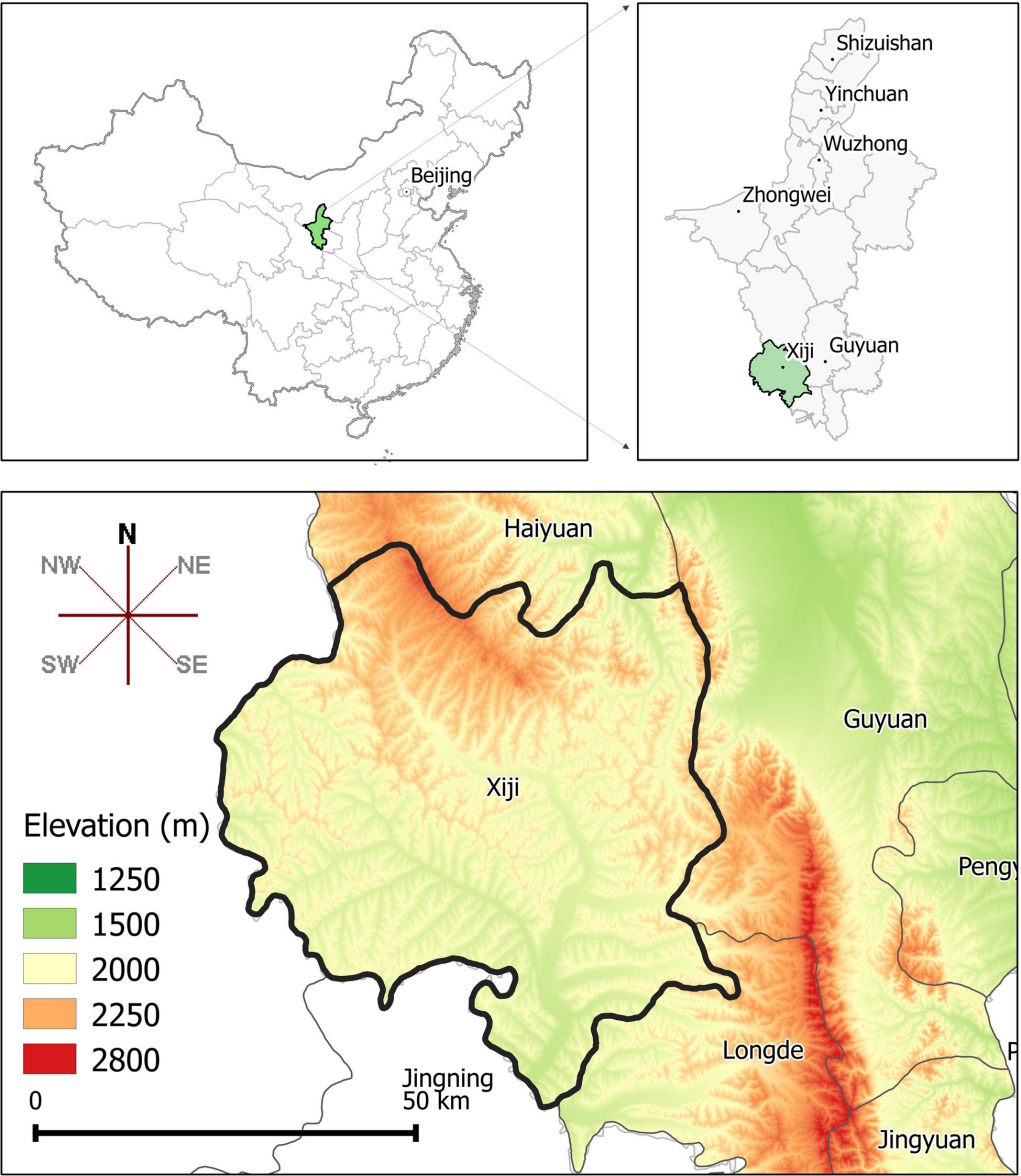
Map and elevation of Xiji County and its location within NHAR, and the location of the Autonomous Region within China

Xiji is a County located in the south of NHAR, between latitudes 35°33' and 36°13'N, and between longitudes 105°20' and 106°4'E. Xiji covers an area of approximately 3985 km^2^ and shares borders with Haiyuan County to the north, Guyuan County to the east, Longde County to the south, and Huining and Jinning Counties that belong to Gansu Province, to the west. Administratively, Xiji is divided into 3 towns and 16 townships, which are then subdivided into 306 villages. In 2015, the total population was 344,045 inhabitants, of whom 58% were of the Hui Islamic ethnic minority and 42% were Han Chinese [36] (Fig. 1).

Xiji lies in a temperate continental monsoon climate zone that is characterized by four distinct seasons. The annual average temperature is 5.37 °C and the average annual precipitation is 418.2 mm. Elevation ranges from 1688 to 2633 m.

Xiji County was selected as the study area because a previous retrospective survey of hospital records conducted in NHAR indicated that high prevalences of human echinococcoses, particularly AE, were concentrated mainly in the southern part of the Autonomous Region, where Xiji is located [37].

### Data on human seropositivity for *E. granulosus* and *E. multilocularis*

**Fig. 2:**
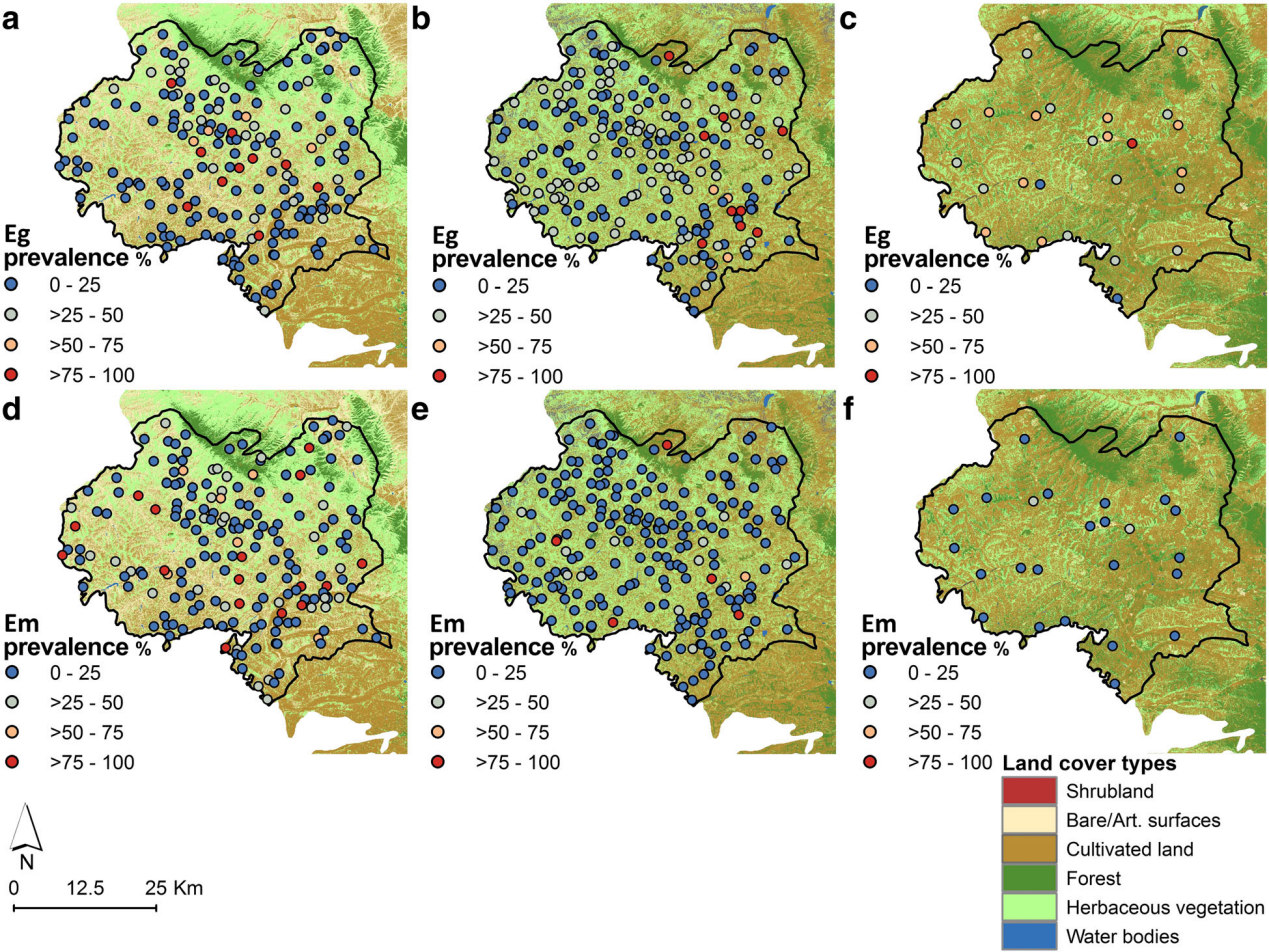
Distribution of school surveys and observed seropositivity for *Echinococcus granulosus* and *E. multilocularis* in 2002–2003 (**a**, **d**), 2006–2007 (**b**, **e**) and 2012–2013 (**c**, **f**) in Xiji County, NHAR, China. A surface of the different land cover types in 2000, 2005 and 2010, respectively, is also presented

Data were obtained from cross-sectional school-based surveys conducted across Xiji County during three distinct time periods: 2002–2003, 2006–2007 and 2012–2013. Surveys were carried out at 190, 219 and 25 locations for each time period, respectively, and included all children aged 6–18 years who agreed to participate (Fig. 2). This age-group was selected in order to ensure that the collected data were representative of recent cases of human exposure.

Exposure information and demographic data were collected with standardised questionnaires that were administered to the students by school teachers. Participants were also asked to provide a small blood sample from the ear lobe for specific antibody testing by enzyme linked immunosorbent assay (ELISA) using *E. granulosus* cyst fluid antigen B (EgB) and *E. multilocularis* crude protoscolex extract (EmP) [38]. Sensitivity of EgB and EmP ELISA is > 85% for CE and > 90% for AE, respectively [23, 38, 39]. Specificity ranges from 70 to 100% for CE [40] and 87% for AE [39]. Finally, abdominal ultrasound was used to screen schoolchildren and detect and classify early CE and/or AE cysts. The World Health Organization classification scheme of CE and AE was used to categorise the hepatic lesions [41–43]. Due to the young age of the study population and the slow rate of growth of the echinococcosis cysts, a very limited of number of ultrasounds showed undefined hepatic changes. Therefore, the results were not included in the statistical models. Participants who were found to be positive for *E. granulosus*, *E. multilocularis* or both were referred to the local health centre for adequate follow-up. Full details of the survey design from 2002 to 2003, data collection and acquisition of ethical approval are reported elsewhere [44]. The survey conducted in 2006–2007 followed the same protocol. A geostatistical design was used to select the schools for the survey in 2012–2013 [45]. A 15 × 15 km grid was created in a geographical information system, and overlaid on the county territory, noting that this survey also covered three other counties (data not presented here). The schools lying in closest proximity to the grid nodes were selected. A secondary set of school located in near proximity to a random subset of those selected at the nodes of the grid (the close pairs) were also selected. This approach has been identified as the most efficient survey design for estimating spatial variability in environmental variables [45].

The geographical coordinates of each school were collected using a hand-held global positioning system. The locations of surveyed schools are shown in Fig. 2. Data collected from the three surveys were combined into a single database.

### Environmental and remotely sensed data

The independent variables included in the analysis were derived from the following data sets: monthly mean temperature and precipitation, elevation, enhanced vegetation index (EVI) and land cover class.

Monthly mean temperature and precipitation data records for the period January 1 1998 to December 31 2013 were provided by the Chinese Academy of Sciences in a raster format at the spatial resolution of 1 km. Data were first collected from 16 local weather stations and interpolated using the Inverse Distance Weighting (IDW) method, but the original weather station data were not available.

Estimates of elevation were obtained from the Advanced Spaceborne Thermal Emission and Reflection Radiometer (ASTER) Global Digital Elevation Model (GDEM) version 2 [46]. The ASTER GDEM is available in the USGS Earth Explorer website in GeoTIFF format at the resolution of 1 arcsecond (approximately 30 m).

Thirty metre resolution Landsat EVI data were obtained from the Earth Resources Observation and Science (EROS) Center Science Processing Architecture (ESPA) On Demand Interface [47]. Data were downloaded annually for the period 1998–2012. To the greatest extent possible, EVI data were acquired from a month between June and November each year for the period 1998–2012. These months correspond to the growing seasons in NHAR. However, acquisition dates varied depending on the availability of the data. When there were no data available for the specified months, the closest-in-time EVI estimates were downloaded for the analyses.

Land cover maps for the years 1996, 2000, 2005, 2010 and 2015 were produced using time-series images retrieved from the Landsat Surface Reflectance Climate Data Record available in Earth Explorer [48]. Six land cover classes were identified: water bodies, artificial surfaces, bare or sparsely vegetated areas, herbaceous vegetation, cultivated land, shrubland and forest (Table 1). Artificial surfaces and bare or sparsely vegetated areas were merged and represented as a single category in the maps and analyses due to significant spectral confusion between them. Details of the original images and the process of land cover classification are provided elsewhere [49].

**Table 1:** Land cover classification scheme and definitions

Land cover type	Description	Content
Water bodies	All areas of water	Streams and canals, lakes, reservoirs, bays and estuaries
Artificial surfaces	Land modified by human activities	Residential areas, industrial and commercial complexes, transport infrastructure, communications and utilities, mixed urban or built-up land and other built-up land
Bare or sparsely vegetated areas	Areas with little or no “green” vegetation present	Dry salt flats, sandy areas, bared exposed rock and mixed barren land
Herbaceous vegetation	Areas characterized by natural or semi-natural vegetation	Grasses and forbs
Cultivated land	Areas where the natural vegetation has been removed/modified and replaced by other types of vegetative cover that have been planted for specific purposes such as food, feed and gardening	Cropland and pasture, orchards, groves, vineyards, nurseries and ornamental horticultural, other cultivated land
Shrubland	Natural or semi-natural woody vegetation with aerial stems less than 6 m tall	Evergreen and deciduous species of true shrubs and trees or shrubs that are small or stunted
Forest	Areas characterized by tree cover or semi-natural woody vegetation greater than 6 m tall	Deciduous forest, evergreen forest and mixed forest

An administrative boundary map of Xiji was downloaded from the DIVA-GIS website [50]. School survey locations were imported into ArcGIS software version 10.3.1 [51] and projected to the Universal Transverse Mercator (UTM) coordinate system zone 48N. Buffer zones of 1 km and 5 km centred on the survey site locations were created using ArcGIS software. All data sets were imported into ArcGIS and linked spatially to the surveyed schools to extract and summarise the environmental data by buffer area.

### Data analysis

Summary statistics were calculated at each location at the time of the survey and at a 5-year lag. A moving 5-year average (MA) was also generated to smooth the estimates of the independent variables. The incorporation of a MA into the analyses allowed assessment of associations over an extended period of time rather than at a single point in time, accounting for the variable latency period of infection. For each location, the summary statistics computed were: (i) annual, summer (June, July and August) and winter (December, January and February) weighted mean series of temperature and precipitation, (ii) spatial mean values of elevation and EVI. The spatial extents (as a percentage of buffer areas) of each land cover category for the years 1996, 2000, 2005, 2010 and 2015 were extracted and used to calculate change rates by buffer area for the periods 1996–2000, 2000–2005, 2005–2010, and 2010–2015. In this way, it was possible to estimate the spatial extent of all land cover classes by buffer area for all years between 1998 and 2012.

The land reform policies and incentive programs to recover degraded land in China might have impacted on landscape fragmentation [52], which could impact on habitat availability for *Echinococcus* spp. intermediate hosts. The five landscape fragmentation metrics that were selected for the analyses were: number of patches (NumP), patch density (PD), mean patch size (MPS), mean shape index (MSI) and edge density (ED) (Table 2). These fragmentation metrics were selected because they provide information about landscape composition, shape, and configuration [53]. These metrics were computed using the Patch Analyst extension of ArcGIS [13].

**Table 2:** Description of the landscape fragmentation metrics that were included in the analyses of human seropositivity for *E. granulosus* and *E. multilocularis* in Xiji County

Metric	Description	Units
Composition		
Number of patches (NumP)	Total number of patches within a buffer	–
Patch density (PD)	Total number of patches per buffer area	/km^2^
Mean patch size (MPS)	Average patch size within a buffer	km
Shape		
Mean shape index (MSI)	Ratio of perimeter to area adjusted by a constant to account for a particular patch shape	–
Configuration		
Edge density (ED)	Amount of edge relative to the buffer area	km/km^2^ (perimeter/area ratio)

### Variable selection

In order to examine separately the association of *E. granulosus* and *E. multilocularis* seropositivities with the environmental factors, univariate logistic regression models were implemented for each parasite exposure using R software version 3.2.2. [54]. Collinearity among all independent variables was assessed using Spearman correlation analyses. If a pair of covariates had a correlation coefficient > 0.9, the variable with the highest value of Akaike Information Criterion (AIC) in the univariate regression models was discarded. Multivariate logistic regression models were developed and the models with the lowest values of AIC were used to select the variables for the final, spatial models. Nonlinear associations between covariates and *E. granulosus* and *E. multilocularis* seropositivities were modelled using quadratic terms, and no interactions were considered.

### Bayesian geostatistical models

Model-based geostatistics was implemented in a Bayesian framework [55] using the OpenBUGS software 3.2.3 rev 1012 [56].

Two distinct models for each of *E. granulosus* and *E. multilocularis* serological status, including parameters for the environmental variables were constructed. The first model (Model I) was developed including the selected explanatory variables for each seropositivity, but without considering the spatial dependence structure of the data; the second model (Model II) assumed that spatial autocorrelation is present in the relative risk of seropositivity. Hence, Model II included the explanatory variables as fixed-effects and a spatially structured random effect. Model fit was compared using the deviance information criterion (DIC), where low DIC values indicate a better model fit. In all analyses, statistical significance was determined with α-levels of 0.05 [as indicated by 95% credible intervals (95% CrI) for odds ratios (OR) that excluded 1].

The complete model, Model II, assumed that *Yi (Yi* = 1 for seropositive schoolchildren and 0 for seronegative schoolchildren) followed a Bernoulli distribution where *Yij* was the serological status of the *i*th child (*i* = 1…5,110) in the *j*th location (*j*= 1…434), and *pij* was the probability an individual *i* being exposed in location *j*, that is,

$$ {Y}_{ij}\sim Bern\left({p}_{ij}\right) $$

$$ \mathrm{logit}\ \left({p}_{ij}\right)={\alpha}_{\varepsilon }+\gamma\ \mathsf{x}\ {\mathrm{age}}_i+\updelta\ \mathsf{x}\ {\mathrm{female}}_i+\sum \limits_{z=1}^z{\beta}_z\ \mathsf{x}\ {\uplambda}_{zj}+{s}_j $$

where *α*_*ε*_ is the survey specific intercept, γ and δ are the coefficients for age and females respectively, *β* is a matrix of z coefficients, *λ* is a matrix of z environmental covariates, and *s*_*j*_ a geostatistical random effect. The spatial correlation structure of the geostatistical random effect was defined by an exponential function of the distance between points:

$$ f\left({d}_{ab};\phi \right)=\exp \left[\hbox{--} \phi {d}_{ab}\right] $$

where *d*_*ab*_ are the distances between pairs of points *a* and *b*, and *ϕ* is the rate of decline of spatial correlation per unit of distance. A normal distribution was specified for the intercept and the coefficients (normal prior with mean = 0 and precision, the inverse of variance, = 1 × 10^-3^). The priors distribution of *ϕ* was uniform with upper and lower bounds set at 0.09 and 100 (the lower bound set to ensure spatial correlation at the maximum separating distance between survey locations was < 0.5). The priors for the precision (1/*σ*_*t*_^*2*^) were specified using a non-informative gamma distribution (with shape and scale parameter values of 0.001 and 0.001, respectively).

A burn-in of 1000 iterations was run and discarded. Subsequent sets of 10,000 iterations were run and examined for convergence. Convergence was determined by visual inspection of history and density plots. The runs were also examined for autocorrelation by visual inspection of the autocorrelation plots. Because autocorrelation was observed for all variables, thinning was applied for subsequent sampling by storing every 10th iteration. Convergence was achieved successfully for all variables in each model at approximately 100,000 iterations. The last 10,000 values from the posterior distributions of each model parameters were stored for the analysis. The rate of decay of correlation between locations (ϕ) with distance and the variance of the spatial component (σ^2^) were also recorded.

The *spatial.unipred* function in OpenBUGS was used for spatial prediction at non-sample locations (defined using a regular 1 × 1 grid overlying the entire Xiji territory). This function applies the model equation at each prediction location using the covariates values extracted for prediction locations and an interpolated value for the geostatistical random effects.

ArcGIS was used to generate maps that represent the posterior distributions of predicted seropositivity for *E. granulosus* and *E. multilocularis* in Xiji County.

## Results

### Sample description

The final data set consisted of 434 school locations and a total of 5110 schoolchildren aged 6–18 years who were screened for human echinococcoses. The surveys involved 845 students in 2002–2003, 2588 in 2006–2007 and 1677 in 2012–2013. The overall seroprevalences of *E. granulosus* and *E. multilocularis* were 33.4 and 12.2%, respectively, ranging from 0 to 100% by school for both parasites. In the first survey, the seroprevalence of *E. multilocularis* among schoolchildren was higher (18.1%) than the seroprevalence of *E. granulosus* (16.8%). However, seropositivity for *E. granulosus* became more common in the second and third survey with seroprevalences of 30.9 and 45.6% compared to seroprevalences of *E. multilocularis* of 12.8% and 8.4%, respectively (Table 3). An abnormal hepatic image compatible with a CE case (0.02% of the total number of schoolchildren in the study) and a query lesion (0.02%) were observed in two different participants in the first survey. Both cases were seropositive for *E. granulosus*. Calcified lesions were also observed in 8 (0.1%) participants in the first survey and 14 (0.3%) participants in the second survey. Among participants with calcifications, 4 (0.01%) were seropositive for *E. granulosus* and 2 (0.03%) were seropositive for *E. multilocularis*. Other asymptomatic liver abnormalities were reported in 4 (0.01%) participants, who were seronegative for both parasite species, in the second survey. The mean age of participants with seropositivity for *E. granulosus* was 12.9 years [median: 13; standard deviation (SD): 2.9], and the mean age for those with seropositivity for *E. multilocularis* was 13.3 years (median: 14; SD: 2.9).

**Table 3:** Human seroprevalence of *Echinococcus granulosus* and *E. multilocularis* infection stratified by gender from three school-based surveys conducted in Xiji County in 2002–2003 (survey 1), 2006–2007 (survey 2) and 2012–2013 (survey 3)

	*E. granulosus*		*E. multilocularis*		Total *n* (%)
	Positive *n* (%)	Negative *n* (%)	Positive *n* (%)	Negative *n* (%)	
Survey 1	142 (16.8)	703 (83.2)	153 (18.1)	692 (81.9)	845 (100)
Survey 2	799 (30.9)	1789 (69.1)	331 (12.8)	2257 (87.2)	2588 (100)
Survey 3	765 (45.6)	912 (54.4)	141 (8.4)	1536 (91.5)	1677 (100)
Total	1706 (33.4)	3404 (66.6)	625 (12.2)	4485 (87.8)	5110 (100)

Figure 2 displays the observed spatial distributions of the seroprevalence of *E. granulosus* and *E. multilocularis* by schools for the three surveys. The maps confirm that seropositivity for *E. granulosus* became more widespread in Xiji County over time, while the distribution of *E. multilocularis* seropositivity became more confined.

### Bayesian geostatistical models

Based on DIC estimates, the Bayesian spatial models (Models II) of seropositivities for *E. granulosus* and *E. multilocularis* were the best-fitting models (Tables 4 and 5). In Model II of *E. granulosus*, girls had a 15.0% (95% CrI: 1.7–29.8%) higher risk of exposure than boys. Also, within the 1 km buffers, there was a 0.7% increase in the odds of seropositivity (95% CrI: 0.4–0.9%) for an increase of 1 mm in summer mean precipitation at the time of the survey, and 6.5% increase (95% CrI: 2.0–10.9%) with 1% increase in water extent at the five-year lag. Forest, shrubland and water coverage in the 5 km buffers were also positively associated with the risk of *E. granulosus*. There were estimated increases of 2.2% (95% CrI: 0.5–3.9%), 194.3% (95% CrI: 44.7–523.1%) and 18.8% (95% CrI: 1.4–38.5%) in the odds of seropositivity for *E. granulosus* for a 1% increase in the extent of forest at the time of the survey, and the extent of shrubland and water at five-year lags. There was a decrease of 2.8% (95% CrI: 0.4–4.8%) in the odds of seropositivity for every year of age. The odds of seropositivity for *E. granulosus* decreased 1.6% (95% CrI: 0.8–2.6%) with a unit increase in NumP, 64.7% (95% CrI: 26.1–82.8%), with 1 km increase in MPS, 6.8% (95% CrI: 4.3–9.3%) with a 1 mm increase in winter mean precipitation and 1.7% (95% CrI: 0.2–3.2%) with a 1% increase in the coverage of bareland/artificial surfaces. In Model II, the variance of the spatially structured random effect increased from 8.4 × 10^4^ (1.6 × 10^4^ to 4.1 × 10^3^) in the first survey to 1.2 × 10^3^ (2.4 × 10^4^ to 4.4 ×10^3^) in the second survey. From this value, the variance decreased to 7.2 × 10^4^ (1.7 × 10^4^ to 2.8 × 10^3^) in the final survey. These findings imply that the amount of spatial variability in the data changed over time with the distribution of seropositive cases becoming more homogeneous at the end of the study period (Table 4).

**Table 4:** Regression coefficients, ORs and 95% CrI from the Bayesian spatial model (Model II) for human seropositivity for *Echinococcus granulosus* in three school-based surveys conducted in Xiji County in 2002–2003, 2006–2007 and 2012–2013

Model	Coefficient, posterior mean (95% CrI)	OR, posterior mean (95% CrI)
α_1_ (Intercept study 1)	-0.23 (-1.79–1.27)	–
α_2_ (Intercept study2)	0.94 (-0.74–2.56)	–
α_3_ (Intercept study 3)	0.38 (-1.10–1.76)	–
Female^a^	0.14 (0.02–0.26)	1.15 (1.01–1.29)
Age	-0.03 (-0.05– -0.01)	0.97 (0.95–0.99)
Summer precipitation same year (1 km)	0.01 (0.00–0.01)	1.01 (1.01–1.02)
EVI same year (1 km)	-5.12 × 10^4^ (-5.10 × 10^4^–4.91 × 10^4^)	0.99 (0.99–1.00)
Cultivated land same year (1 km)	3.24 × 10^3^ (-3.22 × 10^3^–9.94 × 10^3^)	1.00 (0.99–1.01)
Water bodies 5 years prior (1 km)	0.06 (0.02–0.10)	1.06 (1.02–1.10)
Forest same year (1 km)	0.01 (-6.95 × 10^4^–0.02)	1.00 (0.99–1.01)
NumP 5-year average (1 km)	-0.01 (-0.02– -0.01)	0.98 (0.97–0.99)
PD 5-year average (1 km)	1.08 (-0.23–2.83)	2.95 (0.79–16.89)
MPS 5-year average (1 km)	-1.04 (-1.76– -0.30)	0.35 (0.17–0.73)
Winter precipitation same year (5 km)	-0.07 (-0.09– -0.04)	0.93 (0.91–0.95)
Bareland/art surfaces same year (5 km)	-0.02 (-0.03– -0.01)	0.98 (0.96–0.99)
Forest same year (5 km)	0.02 (0.01–0.03)	1.02 (1.01–1.03)
Water bodies 5 years prior (5 km)	0.17 (0.01–0.32)	1.18 (1.01–1.38)
Herbaceous vegetation 5 years prior (1 km)	0.01 (-0.01–0.02)	1.01 (0.99–1.02)
Shrubland 5 years prior (5 km)	1.08 (0.36–1.82)	2.94 (1.44–6.23)
Cultivated land 5 years prior (5 km)	-0.01 (-0.02–0.01)	0.98 (0.97–1.10)
MPS 5 years prior (5 km)	-0.14 (-0.54–0.17)	0.86 (0.58–1.19)
Heterogeneity structured (survey 1)	8.40 × 10^4^ (1.63 × 10^4^–4.12 × 10^3^)	–
Heterogeneity structured (survey 2)	1.18 × 10^3^ (2.42 × 10^4^–4.42 × 10^3^)	–
Heterogeneity structured (survey 3)	7.18 × 10^4^ (1.75 × 10^4^ –2.79 × 10^3^)	–
*ϕ*_*1*_*(*Decay of spatial correlation survey 1)	0.61 (0.04–1.31)	–
*ϕ*_*2*_ (Decay of spatial correlation survey 2)	0.19 (0.03–0.56)	–
*ϕ*_*3*_ (Decay of spatial correlation survey 3)	0.17 (0.02–0.50)	–
DIC	6197	–

*Abbreviations: OR* odds ratio, *95% CrI* 95% credible interval, *DIC* deviance information criterion

^a^Reference category: gender (male)

**Table 5:** Regression coefficients, ORs and 95% CrI from Bayesian spatial model (Model II) for human seropositivity for *Echinococcus multilocularis* in three school-based surveys conducted in Xiji County in 2002–2003, 2006–2007 and 2012–2013

Model/Variable	Coefficient, posterior mean (95% CrI)	OR, posterior mean (95% CrI)
α_1_ (Intercept study 1)	-2.25 (-3.38– -1.39)	–
α_2_ (Intercept study2)	-1.75 (-2.47– -0.94)	–
α_3_ (Intercept study 3)	-2.88 (-3.90– -2.13)	–
Female^a^	0.09 (-0.11–0.24)	1.09 (0.89–1.28)
Age	-0.01 (-0.03–0.02)	0.99 (0.96–1.02)
Summer precipitation same year (1 km)	6.53 × 10^3^ (3.40 × 10^3^–9.31 × 10^3^)	1.01 (1.01–1.02)
EVI same year (1 km)	4.97 × 10^6^ (7.10 × 10^4^–6.21 × 10^4^)	1.00 (0.99–1.00)
Bareland/Art surfaces same year (1 km)	-0.02 (-0.05–0.01)	0.99 (0.99–1.00)
Cultivated land 5 years prior (1 km)	0.01 (-0.01–0.02)	1.01 (0.99–1.02)
Cultivated land same year (1 km)	-0.01 (-0.01–0.01)	0.99 (0.98–1.01)
Herbaceous vegetation 5-year average (1 km)	-0.01 (-0.01–0.01)	0.99 (0.98–1.00)
Water bodies average (1 km)	0.60 (0.24–0.91)	1.82 (1.27–2.50)
Forest same year (1 km)	-0.01 (-0.01–0.01)	1.00 (0.99–1.01)
NumP 5-year average (1 km)	-0.01 (-0.02– -0.01)	0.98 (0.97–0.99)
MPS 5-year average (1 km)	-0.19 (-0.68–0.13)	0.82 (0.50–1.14)
ED 5-year average (1 km)	5.11 × 10^3^ (2.10 × 10^4^–9.97 × 10^3^)	1.01 (1.01–1.02)
Elevation (5 km)	3.99 × 10^4^ (1.64 × 10^3^–1.00 × 10^3^)	0.99 (0.99–1.01)
Winter precipitation 5-year average (5 km)	-0.11 (-0.17– -0.04)	0.89 (0.83–0.95)
Summer temperature 5 years prior (5 km)	-0.01 (-0.38–0.35)	0.99 (0.67–1.42)
Forest 5-year average (5 km)	0.01 (-0.01–0.01)	1.00 (0.99–1.01)
Water bodies 5 years prior (5 km)	0.02 (-0.16–0.20)	1.02 (0.84–1.23)
Water bodies 5-year average (5 km)	-0.02 (-0.07–0.01)	0.97 (0.92–1.01)
Shrubland 5 years prior (5 km)	-1.58 (-2.95– -0.29)	0.20 (0.05–0.74)
Shrubland same year (5 km)	0.95 (-0.45–2.10)	2.59 (0.63–8.23)
Cultivated land same year (5 km)	-0.01 (-0.02–0.01)	0.99 (0.97–1.01)
NumP same year (5 km)	1.66 × 10^4^ (1.25 × 10^4^–4.71 × 10^4^)	1.01 (0.99–1.01)
Heterogeneity structured (survey 1)	3.09 × 10^3^ (5.33 × 10^4^–9.19 × 10^3^)	–
Heterogeneity structured (survey 2)	2.29 × 10^3^ (3.11 × 10^4^–5.28 × 10^3^)	–
Heterogeneity structured (survey 3)	2.29 × 10^3^ (3.11 × 10^4^–5.28 × 10^3^)	–
*ϕ*_*1*_*(*Decay of spatial correlation survey 1)	0.07 (0.01–0.23)	–
*ϕ*_*2*_ (Decay of spatial correlation survey 2)	0.10 (0.02–0.40)	–
*ϕ*_*3*_ (Decay of spatial correlation survey 3)	0.26 (0.09–0.52)	–
DIC	3697	–

*Abbreviations: OR* odds ratio, *95% CrI* 95% credible interval, *DIC* deviance information criterion

^a^Reference category: gender (male)

Model II of *E. multilocularis* seropositivity showed that, within the 1 km buffers, there was an increase of 0.6% (95% CrI: 0.3–0.9%) in the odds of seropositivity for a 1 mm increase in summer mean precipitation. Also, 82.6% (95% CrI: 27.4–150.5%) and 0.5% (95% CrI: 0.02–1.00%) increases in the odds of seropositivity for increases of 1% in the 5-year average of water coverage and 1 km/km^2^ of ED, respectively. The odds of seropositivity for *E. multilocularis* decreased 1.5% (95% CrI: 0.7–2.2%) with a unit increase in NumP, and by 10.6% (95% CrI: 4.6–16.1%) with a 1 mm increase in winter mean precipitation. The odds of seropositivity also decreased 79.4% (95% CrI: 25.8–94.8%) with a 1% increase in the coverage of shrubland. The variance of the spatial random effects decreased from 3.1 × 10^3^ (5.3 × 10^4^ to 9.2 × 10^3^) in survey 1 to 2.3 × 10^3^ (3.1 × 10^4^ to 5.3 × 10^3^) in survey 2 and to 2.3 × 10^3^ (3.1 × 10^4^ to 5.3 × 10^3^) in survey 3.

The values of the decay parameter for spatial correlation (*ϕ)* in the model of *E. granulosus* seropositivity were 0.6 in the first survey, 0.2 in the second survey and 0.2 in the third survey. These estimates indicate that after accounting for the effect of covariates, the radii of the clusters were approximately 555, 1752 and 1959 km, respectively (*ϕ* is measured in decimal degrees, therefore, the cluster size is calculated dividing 3 by *ϕ*; at the equator, one decimal degree is approximately 111 km). The same values in the model of seropositivity for *E. multilocularis* were 0.07, 0.10 and 0.26, for surveys 1, 2 and 3, with cluster sizes of 4757, 3330 and 1280 km, respectively. These results imply that spatial correlation in the risk of seropositivities for *E. granulosus* and *E. multilocularis* was evident between schools with relatively large distances separating them.

### Spatial predictions

**Fig. 3:**
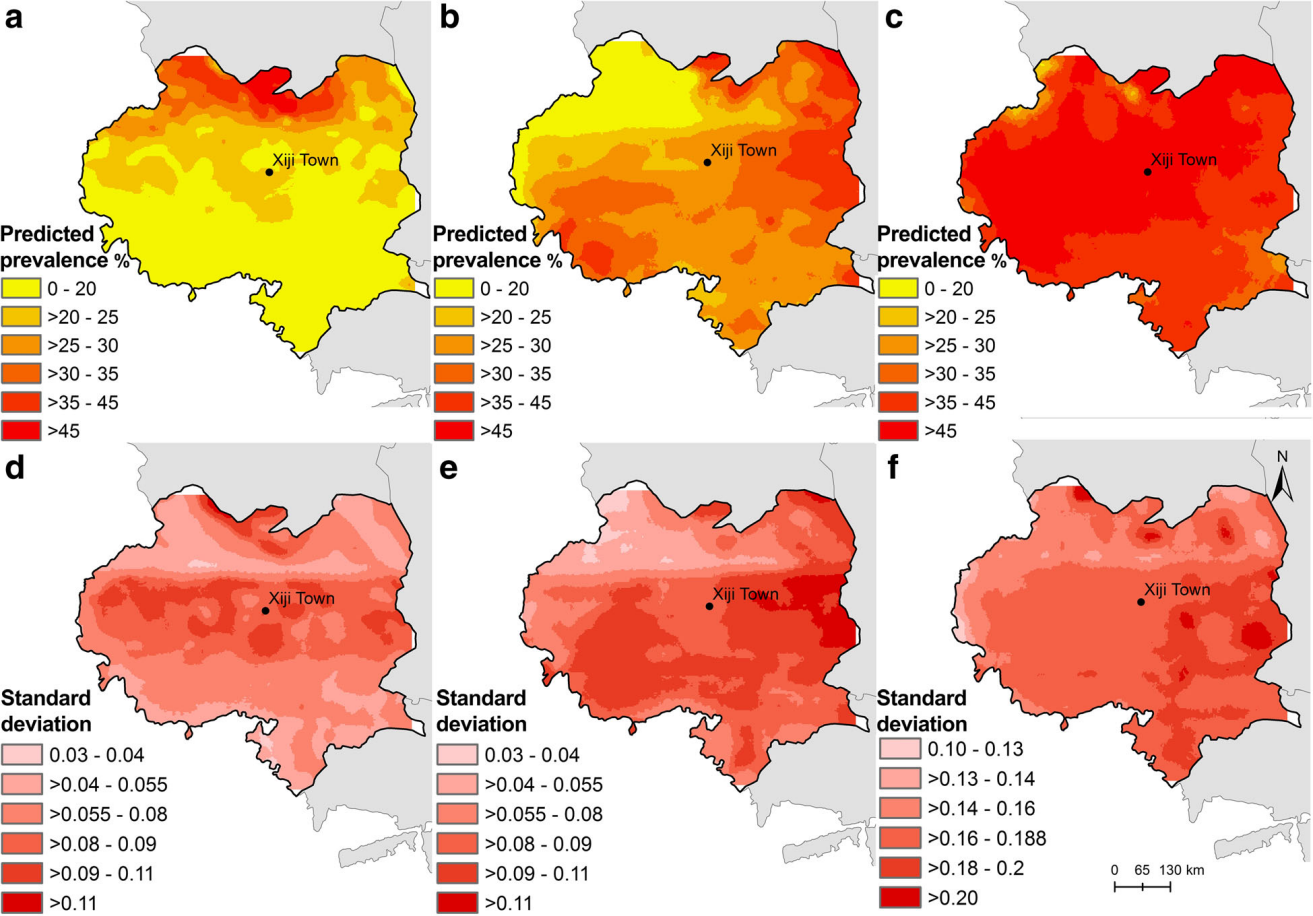
Spatial distribution of predicted seropositivity for *Echinococcus granulosus* in schoolchildren aged 6–18 years and standard deviations in 2002–2003 (**a**, **d**), 2006–2007 (**b**, **e**) and 2012–2013 (**c**, **f**) in Xiji County, NHAR, China

Maps of the median and SD of the posterior distributions of predicted seroprevalence of *E. granulosus* for the years 2002–2003, 2006–2007 and 2012–2013 are shown in Fig. 3. The north-central part of the county was an area with persistent high predicted seroprevalence during the surveys, with the range of high seroprevalence areas expanding to cover the entire county by the time of the third survey. Prediction uncertainty was generally higher in the central and eastern parts of the county.

**Fig. 4:**
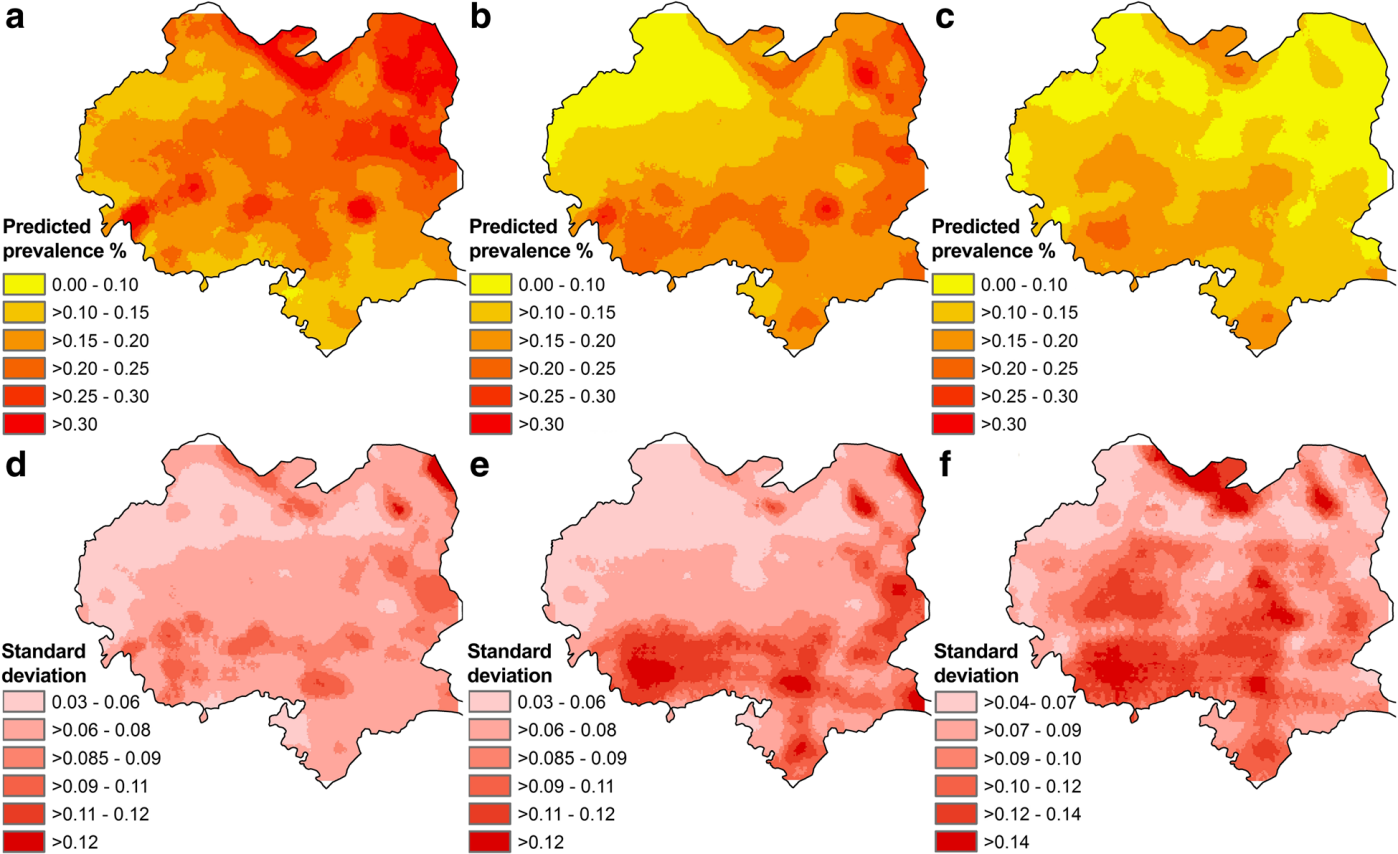
Spatial distribution of predicted seropositivity for *Echinococcus multilocularis* in schoolchildren aged 6–18 years and standard deviations in 2002–2003 (**a**, **d**), 2006–2007 (**b**, **e**) and 2012–2013 (**c**, **f**) in Xiji County, NHAR, China

Maps of the median and SD of the posterior distributions of predicted seroprevalence of *E. multilocularis* are presented in Fig. 4. Areas of high predicted seroprevalence in the north, northeast and centre of the county gradually decreased from survey 1 to survey 3, leaving some residual foci of high seroprevalence in the central north and southwest parts of the county. Maps of the posterior SDs demonstrate that the level of uncertainty increased over time.

## Discussion

In this study, we present model-based predictive risk maps of human seropositivities for *E. granulosus* and *E. multilocularis* for Xiji County, for the years 2002–2003, 2006–2007 and 2012–2013. Previous epidemiological reports on CE and AE infections in NHAR were mostly descriptive, reporting prevalence estimates at specific locations [44, 57, 58]. Spatially explicit statistical models were constructed previously to predict the spatial distribution of infection with *E. multilocularis* among the non-student population in Xiji County in 2002–2003 [27]. That model showed that the landscape features associated with an increased AE risk in Xiji County differed from previous observations in Zhang County in the neighbouring Gansu Province [21, 23]. Unlike the findings in Zhang County, where grassland/shrubland favoured the creation of optimal peri-domestic habitats for *E. multilocularis* intermediate host species, and the development of a peri-domestic cycles involving dogs [21, 23], in Xiji County, abundance of reforested lowland pastures was correlated with higher prevalence of human AE risk. This finding supports the hypothesis that the transmission of *E. multilocularis* may occur through a diversity of host communities in China [27]. Therefore, extended monitoring of the seroprevalence of both, CE and AE, in the context of landscape transformation was suggested for Xiji County to assess the potential impact of local environmental factors on the transmission dynamics of *E. granulosus* and *E. multilocularis* [27]. Also, predictive estimates of the prevalence of infections in humans over time are currently required to inform and support the ongoing implementation process for prevention and control [10, 29].

In general, the risk of seropositivity for *E. granulosus* expanded in Xiji County over the study period. In 2002–2003, *E. granulosus* risk was clustered mainly in the north-central part of Xiji, an area that corresponds largely to the Yueliang mountain range (2626 m), and where predominant vegetation consist of forest, grassland and cultivated land [49]. *Echinocuccus granulosus* risk expanded towards the east in 2006–2007 and decreased in the north-west. Finally, the risk of seropositivity was between 35 and 45% in almost the entire county territory in 2012–2013. These findings concur with reports of the apparently expanding geographical range of *Echinococcus* spp. [16, 59–64]. In Xiji County, livestock and arable agriculture are common practices among most local communities and represent higher risk of *Echinococcus* spp. exposure. Therefore, intensification in livestock production to supply the growing demand for resources may have pushed the local human settlements into close proximity with their livestock and the habitats of other potential *Echinococcus* spp. hosts [65]. According to data from the Gridded Livestock of the World v.2.0, in 2006, sheep and cattle populations were distributed in the entire territory of Xiji County with higher densities, 20–50 and 10–50 heads per square kilometre, respectively, in the north-west [65]. The prevalence of CE in sheep was estimated to be 52% in NHAR in 2008, and between 0–9% according to more recent studies conducted in local areas no larger than counties [66–68]. These prevalence estimates may have varied due to local or individual conditions that facilitated high transmission within patches of CE endemicity. Also, studies conducted at the provincial level have found that 81% of cattle, 3% of goats, 19% of camels and 24% of pigs were infected with *E. granulosus* in 2008 [69].

The land cover in NHAR has been modified considerably in recent decades [49, 70]. Because landscape characteristics may determine directly or indirectly the feeding behaviour, growth rates, reproductive efficiency and immunological mechanisms of domestic animals [71], it was not surprising to find that the extent of various vegetation types were associated with the risk of seropositivity for *E. granulosus*. A reduction of bareland and the increases of woody vegetation types such as forest and shrubland may have sustained the *E. granulosus* life-cycle by facilitating the geographical expansion and interactions of competent hosts that move in response to available food sources [8, 72]. The movement of domestic animals and changes in their feeding practices can also be explained by land cover changes that contributed to loss or fragmentation of natural habitats indicated by metrics such as, NumP and MPS, that were significantly associated with the risk of seropositivity for *E. granulosus* [73–75]. The positive association between the seroprevalence of *E. granulosus* and the extent of area covered by water was unexpected and deserves further investigation. However, this relationship may be explained partially by the same mechanism that associates positively and negatively *E. granulosus* risk with summer and winter precipitation, respectively, at the time of the survey. Sufficient ground moisture is an important determinant of the survival and infectivity of *Echinococcus* spp. eggs in the external environment [76, 77]. Also, due to the lack of piped water in some areas in the south of NHAR in past decades, the inhabitants had to rely mainly on natural drinking water supplies such as seasonal rivulets and temporary wells dug in dry-river beds [78]. Domestic dogs had also free access to these water supplies, which may have led to water contamination with the parasite eggs and increased risk of *Echinococcus* spp. transmission to the human population [78].

Increased annual rainfall has been shown to be associated with high infection rates of *E. granulosus* in livestock from hyperendemic regions for CE in Ethiopia and north-central Chile [79, 80]. Also, studies conducted in Iran and Saudi Arabia reported seasonal variations in the prevalence of *E. granulosus* infection during abattoir meat inspections [81, 82].

The observed differences of *E. granulosus* risk among females and males and the negative association with age may be exposure-related [44, 83, 84]. However, it has also been suggested that immunological and hormonal gender differences may account for higher infection rates in females than males [44].

In contrast to the high seroprevalence and geographical expansion of the seropositivity for *E. granulosus*, the seropositivity for *E. multilocularis* was lower and decreased during the three surveys. In 2002–2003, most areas in the county had estimated seroprevalences of *E. multilocularis* between 10 and 30%, with higher risk in those communities located in the north-east and central part. An important reduction was observed in the north-western area of Xiji in 2006–2007, and in north-eastern Xiji in 2012–2013. Seroprevalences of *E. multilocularis* remained highest in the south-west throughout the surveys. Overall, the findings of this study do not support the evidence from Europe and other regions in Asia that indicates the spreading of *E. multilocularis* [2, 85, 86]. This discrepancy could be due to different local transmission dynamics of the parasite in Xiji County and to novel interactions between the recently transformed local landscape, the parasite and its hosts [27]. However, issues related to the inherent limitations of sampling variation and different methodological approaches should also be considered.

Landscape change and fragmentation have been identified as important determinants of the population dynamics of several species of wild mammals that are common intermediate host of *E. multilocularis* [87–90]. In eastern France, population outbreaks of *Microtus arvalis* and *Arvicola terrestris* were reported in areas where ploughed fields were converted into permanent grassland [21, 22]. Significant positive associations of *E. multilocularis* infection in humans and foxes with the extent of grassland were also reported in the same region [21, 91, 92]. The distribution of small mammals also varied in response to the transient augmentation of grassland/shrubland that followed a period of deforestation in Gansu Province [21, 23], and to overgrazing and fencing practices in the north-western part of Sichuan Province on the Tibetan Plateau [24–26]. Recently, it was demonstrated that low-biomass degraded grassland habitats influence the presence of *Ochotona* spp. in Serxu County, Sichuan Province [28]. In NHAR, the diversity of small mammal assemblages was related to afforestation, and was lower than that of assemblages in areas where deforestation occurred [93]. Lowland pastures that were described as heavily grazed grassland interspersed with forest or shrub cover were associated with higher prevalence of human AE [27]*.* The results of this study showed significant associations with fragmentation metrics and seropositivity for *E. multilocularis*. It was also found that shrubland did not provide an optimal habitat for the transmission of *E. multilocularis*. Because different classification methods and definitions were used in relation to the previous study in Xiji County, the results need to be interpreted with caution. However, the results support the hypothesis that the land cover characteristics facilitating *E. multilocularis* transmission in Xiji County are different from those favouring the transmission of the parasite in the south of Gansu Province [27]. Despite many epidemiological differences between *E. granulosus* and *E. multilocularis*, the significant positive associations between *E. granulosus* risk and the extent of water and summer precipitation, and the negative association with winter precipitation, were also found for *E. multilocularis* risk in Xiji County. Examination of the viability of *E. multilocularis* eggs particularly, has revealed that the eggs are sensitive to microclimatic conditions such as moisture levels or humidity [76]. Laboratory studies indicated that *E. multilocularis* eggs are more resistant to heat if suspended in water [94].

Interventions to reduce the risk of human infection in NHAR are in line with the guidelines of the National Control Programme [6]. Mass-community screening surveys, health education campaigns, regular dog treatment with praziquantel, patient treatment and animal offal inspection and control in slaughterhouses have been taking place across the NHAR since 2005 [6, 7]. Due to the lack of surveillance data and an incomplete understanding of the factors influencing parasite transmission, it has been difficult to forecast the impacts of the control measures [10]. The results of the current study are important for estimating the burden of CE and AE in Xiji County. In addition, considerable small-scale spatial variation in seropositivities for *E. granulosus* and *E. multilocularis* was observed which indicates that there is scope for predictive risk maps to help inform spatially targeted control measures in Xiji County. Areas of priority for AE control include the north and south-western part of the county, whereas CE control is required throughout.

Important limitations of the study were the different survey designs used between periods, affecting comparability of the data, and the use of schools to geolocate children, which might not reflect where exposure occurred. Also, children seropositivity for *E. granulosus* and/or *E. multilocularis* was defined using specific antibody testing by ELISA using EgB and EmP. The poor diagnostic performance of these current serological tests and cross-reaction with other helminthic infections, including other types of human echinococcoses [95], and gastrointestinal malignancies remain a critical issue for the diagnosis of CE and AE and represent a source of misinterpretation in areas where both infections co-exist [35, 90]. Nevertheless, the analyses revealed that *E. granulosus* risk has increased and become more widespread across Xiji County during the study period. The patterns of *E. multilocularis* risk did not concur with the reported expansion of *E. multilocularis* in other regions. Clearly, control of CE is a public health priority in Xiji County, whereas further research is required to explore in more detail the potential factors that may be influencing the changing burden of AE.

## Conclusions

This work provides detailed geographical information regarding the changes in the predicted prevalence of human seropositivities for *E. granulosus* and *E. multilocularis* in Xiji County, a highly endemic area for human echinococcoses. The study period was from 2002 to 2013, during which extensive landscape restoration projects were implemented in NHAR and other parts of China. The different models developed in this study indicate that the human seropositivity for *E. granulosus* expanded across Xiji during the study period, while seropositivity for *E. multilocularis* became more confined in communities located in the south of the county. These results help to identify priority areas where targeted prevention and control efforts are most required.

## Abbreviations

95% CrI: 95% credible intervals
AE: Alveolar echinococcosis
AIC: Akaike information criterion
ASTER: Advanced Spaceborne Thermal Emission and Reflection Radiometer
CE: Cystic echinococcosis
DALYs: Disability-adjusted life years
DIC: Deviance information criterion
ED: Edge density
EgB: Cyst fluid antigen B
EmP: *E. multilocularis* crude protoscolex extract
ELISA: Enzyme-linked immunosorbent assay
EROS: Earth Resources Observation and Science
ESPA: Center Science Processing Architecture
EVI: Enhanced vegetation index
GDEM: Global digital elevation model
IDW: Inverse distance weighting
MA: Moving average
MPI: Mean shape index
MPS: Mean patch size
NHAR: Ningxia Hui Autonomous Region
NUMP: Number of patches
OR: Odds ratio
PD: Patch density
SD: Standard deviation
USGS: United States Geological Survey
UTM: Universal Transverse Mercator
